# Perspective on the nursing management for gestational diabetes mellitus: A perspective

**DOI:** 10.1097/MD.0000000000041862

**Published:** 2025-03-21

**Authors:** Ya-ting Fan, Xin-hui Wang, Qing Wang, Xiao-tong Luo, Jing Cao

**Affiliations:** a Department of Obstetrics, Baoji People’s Hospital, Baoji, China.

**Keywords:** gestational diabetes mellitus, management, nursing care, perspective, prevention

## Abstract

This study provides a comprehensive examination of gestational diabetes mellitus (GDM), shedding light on the geographical and ethnic variations in its prevalence. It elucidates the diagnostic evolution, noting the transition from rudimentary glucose tests to the more sophisticated Oral Glucose Tolerance Test (OGTT), which not only facilitates early detection but also standardizes screening protocols. The study delves into the evolution of GDM diagnosis, emphasizing the standardization of the OGTT and its pivotal role in enhancing early detection rates. It meticulously discusses holistic management approaches for GDM, encompassing tailored dietary interventions, prescribed physical activity, and pharmacotherapy. The need for individualized strategies to optimize glucose control is strongly emphasized. The study underscores the significance of mental health in GDM management, advocating for integrated psychological support and stress management interventions to bolster metabolic regulation. An exploration of telemedicine and artificial intelligence highlights their potential to revolutionize GDM care by enabling real-time monitoring and personalized interventions, thus improving patient outcomes. An analysis of health policies and educational efforts underscores their impact on GDM management, advocating for proactive measures to mitigate its prevalence through public health initiatives. The study identifies key research gaps and offers a focused analysis of critical advancements in GDM management, including personalized care strategies and the role of innovative technologies such as artificial intelligence and telemedicine in improving outcomes. Finally, the study calls for further research into personalized treatment modalities and innovative diagnostic tools to address existing gaps in GDM management, particularly in diverse demographic groups.

## 1. Introduction

Gestational diabetes mellitus (GDM) is defined as a form of glucose intolerance that is first detected during pregnancy.^[1]^ It is commonly diagnosed in the later stages of pregnancy and presents significant health risks to both the mother and fetus.^[2]^ GDM is a pivotal concern within obstetrics, gynecology, and global public health due to its prevalence and impact.^[3]^ The incidence of GDM exhibits notable variations across different regions, influenced by factors such as geographic location, ethnicity, socioeconomic status, and genetic predispositions.^[3]^ According to the World Health Organization, the prevalence of GDM is higher in regions like the Middle East and South Asia. Conversely, some Western countries report lower prevalence rates, attributed to more systematic approaches to prevention and screening. Additionally, the global rise in obesity and lifestyle shifts are contributing to an increased incidence of GDM, particularly in low- to middle-income countries.^[4]^

The study of GDM is crucial not only for understanding its pathophysiological underpinnings but also for assessing its long-term effects on the health of mothers and their children.^[5]^ Research into GDM helps uncover effective strategies for prevention and management, which are vital for reducing the incidence of pregnancy-related complications and subsequent chronic conditions.^[6]^ Clinical evidence suggests that without proper management, GDM can lead to increased risks of pregnancy complications such as preeclampsia and preterm birth. It may also predispose women to develop type 2 diabetes mellitus (T2DM) and cardiovascular diseases after pregnancy. The offspring of women with GDM are at an increased risk of developing conditions like obesity and T2DM later in life.^[2,7]^

This study aims to provide a thorough analysis of the latest research developments in GDM. It will cover aspects such as screening processes, clinical management, and the broader impacts of GDM on maternal and child health. To narrow the scope and emphasize the most critical areas of research and management, this study focuses on recent advancements in diagnostic criteria, personalized management strategies, and the integration of emerging technologies. These contributions aim to provide actionable insights for clinicians, guide future research directions, and inform public health strategies tailored to diverse populations.

## 2. Search strategy and selection criteria

A comprehensive and systematic search was conducted to identify relevant literature on nursing management for GDM. The search utilized key terms, including “nursing management,” “nursing care,” “nursing intervention,” “diabetes mellitus,” and “gestational diabetes mellitus.” Databases searched included China National Knowledge Infrastructure and PubMed, covering publications from inception to 2024.

### 2.1. Inclusion criteria

Studies were eligible for inclusion if they met the following criteria:

1. Focused on nursing management strategies for GDM.
2. Presented clinical outcomes or experimental evidence evaluating the effectiveness of nursing interventions.
3. Were peer-reviewed and published in English or Chinese languages.

### 2.2. Exclusion criteria

Studies were excluded if they:

1. Addressed interventions unrelated to nursing management for GDM.
2. Were duplicate publications or not relevant to the topic of nursing management for GDM.

### 2.3. Study selection

The selection process adhered to the predefined inclusion and exclusion criteria. Two independent authors assessed the studies, and any disagreements were resolved by a third author through discussion to ensure objectivity and consistency.

## 3. Risk factors and diagnosis of GDM

GDM is a condition characterized by high blood sugar levels that develop during pregnancy and can adversely affect both the mother and the fetus.^[5]^ Effective management and early intervention are critical and depend heavily on the accurate identification and handling of associated risk factors.

### 3.1. Main risk factors

The development of GDM is linked to a combination of genetic, lifestyle, and historical health factors.^[8–10]^ Genetic predisposition plays a crucial role, as evidenced by higher incidence rates in women with a family history of GDM. Lifestyle factors such as diets high in calories, limited physical activity, and excessive body weight significantly contribute to the risk. Additionally, a personal history of GDM or conditions like polycystic ovary syndrome also serve as significant predictors of GDM recurrence.

### 3.2. Evolution of diagnostic criteria

Over the years, the criteria for diagnosing GDM have evolved to align with advances in medical research and changes in population health metrics.^[1,11]^ Initially reliant on random glucose testing and symptomatic analysis, the methodology shifted towards a more reliable and quantifiable measure with the adoption of the Oral Glucose Tolerance Test (OGTT) in the 1960s. Recent updates by leading health organizations have refined these criteria further, advocating for early and standardized screening processes to include OGTT between the 24th and 28th weeks of gestation.

### 3.3. Optimization of screening techniques

While the OGTT remains the benchmark for GDM diagnosis, its complexity and the discomfort it causes have led to research into alternative methods.^[12–14]^ Technologies such as Continuous Glucose Monitoring Systems offer a less invasive, more patient-friendly approach with the potential for real-time blood glucose monitoring. Additionally, research into biomarkers specific to GDM is exploring avenues for earlier and more precise detection. These innovations aim to enhance the screening process, making it more accessible and tolerable for expectant mothers, thereby facilitating earlier interventions.

By sharpening the focus on these areas, research continues to advance our understanding and management of GDM, aiming to mitigate its impact on pregnancy outcomes and long-term health for mothers and their children. These efforts not only improve clinical practices but also aim to reduce the prevalence and severity of GDM through targeted preventive measures and more personalized care strategies.

## 4. Management strategies for GDM

### 4.1. Nutritional management

In managing GDM, dietary interventions are critical. Recent studies have shown that strategically modifying the diet can profoundly influence glycemic control^[15–17]^ (Table 1). Recent systematic reviews and meta-analyses affirm the efficacy of dietary interventions, particularly low-glycemic index (GI) diets, in managing GDM^[18,19]^ (Table 1). These interventions have been shown to significantly reduce fasting glucose levels and improve postprandial glycemic responses, thereby diminishing the reliance on pharmacological treatments.^[18,19]^ A meta-analysis published in 2016 further highlighted that adopting a low-GI diet enhances maternal glucose regulation while presenting a safe and effective noninvasive approach for optimizing pregnancy outcomes.^[19]^ It is recommended to prioritize the consumption of foods with a low glycemic index to minimize fluctuations in blood glucose levels and to limit intake of high-sugar and high-fat foods.^[15]^ Implementing a meal structure that involves multiple small meals throughout the day rather than fewer large meals has been found to be particularly effective in maintaining stable glucose levels. Diet plans should be customized for each patient by a qualified dietitian based on individual factors such as body weight, blood glucose readings, and the gestational stage.^[16]^

**Table 1:** Summary of included studies

Reference	Publication type	Patients	Treatment/intervention	Main findings
Moses RG et al, 2009^[15]^	Randomized trial	Pregnant women with GDM	Low-glycemic index diet	Low-glycemic index diet reduced insulin needs in GDM patients.
Zhang C et al, 2006^[16]^	Prospective cohort study	Women assessed for GDM risk	Dietary fiber and glycemic load	Higher dietary fiber intake reduced GDM risk; high glycemic load increased it.
Bao W et al, 2013^[17]^	Prospective cohort study	Women assessed for GDM risk pre-pregnancy	Dietary protein intake	High animal protein intake increased GDM risk; plant-based protein reduced risk.
Oostdam N et al, 2011^[18]^	Systematic review and meta-analysis	Pregnant women at risk for GDM	Various GDM prevention interventions	Interventions like diet and exercise reduced GDM incidence.
Wei J et al, 2016^[19]^	Meta-analysis	Pregnant women with GDM	Low-glycemic index diets	Low-glycemic index diets improved glycemic control in GDM patients.
Russo LM et al, 2015^[20]^	Systematic review and meta-analysis	Pregnant women assessed for GDM risk	Physical activity interventions	Physical activity reduced GDM risk and improved outcomes.
Langer O et al, 2000^[21]^	Randomized trial	Pregnant women with GDM	Comparison of glyburide and insulin	Glyburide and insulin were similarly effective for GDM management.
Ijas H et al, 2011^[22]^	Prospective randomized study	Pregnant women with GDM	Metformin treatment	Metformin was effective and safe for GDM management.
Huizink AC et al, 2004^[23]^	Psychological study	Pregnant women with prenatal stress	Prenatal stress assessment	Prenatal stress increased risk of psychopathology in offspring.
Kim C et al, 2005^[24]^	Observational study	Women with GDM and pregnancy-induced hypertensive disorders	Health status monitoring	Health status changes linked to GDM and hypertensive disorders.

GDM = gestational diabetes mellitus.

### 4.2. Physical activity recommendations

Physical activity serves as a cornerstone in the management of GDM.^[20]^ A recent meta-analysis of randomized controlled trials demonstrated that moderate-intensity exercise substantially improves insulin sensitivity while significantly lowering the risks of macrosomia and preterm birth in women with GDM^[20]^ (Table 1). These findings underscore the critical role of structured exercise programs as an integral component of evidence-based care protocols, reinforcing their value in both maternal and neonatal health. According to the latest guidelines, unless contraindicated, pregnant women should engage in at least 150 minutes of moderate-intensity aerobic activity per week, such as walking or swimming. Longitudinal studies provide compelling evidence that consistent adherence to exercise guidelines yields substantial postpartum benefits, including enhanced maternal cardiovascular health and a reduced lifetime risk of type 2 diabetes for both mothers and their offspring. This highlights the dual impact of regular physical activity on immediate glycemic control during pregnancy and the long-term prevention of chronic metabolic conditions. Such consistent, moderate exercise has been demonstrated to improve insulin sensitivity and thus enhance blood glucose management. It is imperative that any exercise regimen be conducted under medical supervision to ensure both the efficacy and safety of the physical activities recommended.

### 4.3. Pharmacological treatment

Pharmacological intervention becomes necessary when lifestyle modifications alone do not suffice to manage blood glucose levels effectively^[21,22]^ (Table 1). Insulin therapy remains the treatment of choice for GDM due to its safety profile, as it does not cross the placental barrier, thereby avoiding any direct pharmacological impact on the fetus. The exploration of novel oral hypoglycemic agents, like metformin, has expanded; however, their application in pregnancy should be approached with caution and under strict medical supervision. Treatment plans involving medications should be distinctly tailored by specialists in endocrinology or obstetrics, considering the patient’s specific health parameters.

### 4.4. Psychological and behavioral health

Maintaining mental and behavioral health is essential in the comprehensive management of GDM^[23,24]^ (Table 1). Research indicates that psychological stress during pregnancy can adversely impact glycemic control.^[23]^ Effective management thus necessitates the provision of professional psychological support and techniques for managing stress. Services tailored to the psychological needs of patients with GDM should address issues such as anxiety and depression and may include psychological counseling and therapeutic interventions. Enhancing support networks through group therapy or support groups for pregnant women can also significantly contribute to improved mental well-being.

Each of these strategies must be adapted and implemented based on individual patient needs and under the guidance of an interdisciplinary team of healthcare professionals to optimize outcomes in the management of GDM.

## 5. Long-term health impacts of GDM on mothers and offspring

### 5.1. Maternal health consequences

Increased risk of T2DM: Women with prior GDM experience are at a heightened risk of transitioning to T2DM in the postpartum period. This risk remains significantly higher compared to women without a GDM history, underscoring the need for ongoing glucose monitoring and lifestyle management post-pregnancy.^[6]^

Cardiovascular disease: There is a demonstrable increase in the incidence of cardiovascular conditions, including hypertension and coronary artery disease, in women post-GDM.^[25]^ Recent longitudinal studies have established that women with a history of GDM face a 43% increased risk of myocardial infarction within 10 years postpartum, even after accounting for confounding lifestyle factors.^[25]^ This evidence underscores the necessity for sustained cardiovascular monitoring and early preventive interventions in this high-risk group. These findings suggest that GDM may share common pathophysiological pathways with metabolic syndrome, which merits further investigation.

### 5.2. Offspring health outcomes

Birth complications and macrosomia: Infants born to mothers with GDM have a higher propensity for being macrosomic, a condition that complicates the delivery process and increases the risk of birth injuries such as shoulder dystocia.^[2,26,27]^

Obesity and metabolic risks in offspring: Children of mothers with GDM are predisposed to obesity and metabolic syndromes in later life, including an elevated risk of developing T2DM and cardiovascular diseases. A systematic review confirmed that maternal GDM significantly increases the risk of early-onset metabolic syndrome in offspring.^[28]^ Notably, lifestyle interventions initiated during pregnancy demonstrated efficacy in mitigating these risks, emphasizing the intergenerational impact of GDM and the importance of proactive maternal health strategies. This intergenerational transmission of health risks highlights the critical need for monitoring and early lifestyle interventions.

Neurodevelopmental and behavioral effects: Preliminary evidence suggests a potential association between maternal GDM exposure and neurodevelopmental disorders in offspring, such as Attention-deficit hyperactivity disorder. However, these findings necessitate further research to clarify causal pathways and potential mechanisms.

### 5.3. Prevention and management

Lifestyle modifications: Preventive strategies primarily focus on dietary adjustments and enhanced physical activity to mitigate the risk of GDM.^[29–31]^ These interventions not only aim to prevent the onset of GDM but also contribute to overall health improvements during and after pregnancy.

Challenges in implementation: The effectiveness of these preventive measures often encounters obstacles such as varying levels of adherence to lifestyle changes, continuity of care postpartum, and the influence of socioeconomic factors on health behaviors.

Screening and early intervention: Targeted screening for GDM risk factors and early intervention for those identified at high risk are essential.^[30]^ Effective screening requires robust risk assessment models and access to comprehensive healthcare services to manage the condition effectively.

In summary, GDM poses significant long-term health risks to both mothers and their children, necessitating rigorous preventive and management strategies. These strategies should integrate lifestyle interventions with continuous medical follow-up and interdisciplinary approaches to address the multifaceted nature of GDM and its consequences.

## 6. Technological innovations in GDM management

### 6.1. Telemedicine and digital health solutions

Telemedicine has emerged as a pivotal component in managing GDM, leveraging digital communication tools to facilitate remote monitoring and management.^[32]^ Patients are now able to perform self-monitoring of blood glucose levels at home and transmit this data in real-time to healthcare providers. Telemedicine platforms enable real-time interaction between patients and healthcare providers, allowing dynamic adjustments to treatment plans based on continuous data feedback.^[32]^ Recent advancements in telemedicine have seen the incorporation of sophisticated platforms that combine real-time monitoring with predictive analytics, leveraging machine learning algorithms to anticipate complications and optimize care. A study reported a great reduction in hospital admissions for patients with GDM using telemedicine interventions, highlighting their role in enhancing preventive care and improving clinical outcomes.^[33]^ This approach enhances personalized care by promptly addressing glucose level fluctuations, thereby reducing the risk of complications associated with delayed interventions. Additionally, telemedicine expands access to specialized care, particularly for patients in remote or underserved regions, effectively mitigating geographical disparities. This capability not only allows for immediate adjustments in therapeutic strategies based on fluctuating glucose levels but also enhances patient engagement and adherence by integrating health management into daily life. Mobile health applications augment this process by providing tailored dietary and exercise recommendations, thus enhancing lifestyle management and overall treatment adherence.

### 6.2. Artificial intelligence in clinical decision making

Artificial Intelligence (AI) plays a transformative role in GDM management by analyzing extensive datasets to identify patterns and predict outcomes.^[34]^ Utilizing machine learning algorithms, AI systems can assess risk levels, anticipate complications, and customize treatment regimens. These systems analyze historical health data and continuous monitoring metrics to provide clinicians with precise treatment recommendations. AI technologies play a pivotal role in predictive modeling, enabling the identification of patients at heightened risk for complications such as preterm birth or severe hyperglycemia.^[35]^ AI systems have recently evolved to integrate continuous glucose monitoring data with patient-reported outcomes, providing dynamic and precise insights into glycemic variability.^[36]^ A review demonstrated that AI-powered tools improved glycemic control greater compared to traditional methods, emphasizing their transformative potential in delivering tailored and efficient care for GDM patients.^[36]^ Machine learning algorithms analyze complex datasets, including patient histories, to predict adverse outcomes, empowering clinicians to take preemptive actions. Moreover, AI-driven decision support systems enhance clinical precision by optimizing insulin dosages and tailoring dietary recommendations to individual metabolic profiles, resulting in improved glycemic control and overall patient outcomes. For example, AI algorithms can determine optimal insulin doses or dietary changes based on individual patient profiles, thereby enhancing the accuracy and effectiveness of medical interventions.

### 6.3. Wearable technology for continuous monitoring

Wearable devices have become increasingly significant in GDM management, providing continuous tracking of vital health parameters such as glucose levels, heart rate, and physical activity.^[37,38]^ These devices facilitate a dynamic approach to treatment by offering ongoing insights into a patient’s health status, which can be crucial for timely adjustments to treatment plans. The continuous data stream helps create a comprehensive health profile for each patient, enabling personalized and dynamic management plans that adapt to each patient’s unique health trajectory. The integration of wearable devices with AI analytics offers deeper insights into maternal and fetal health by synthesizing data from glucose monitors, physical activity trackers, and dietary logs.^[37]^ This holistic perspective facilitates a comprehensive evaluation of patient adherence and progress, enabling the implementation of more targeted and effective interventions. These advancements illustrate the synergistic potential of AI and wearable technology in transforming the management of GDM.

### 6.4. Integration and impact on healthcare

The integration of these technologies transforms traditional care models by enhancing the precision and personalization of diabetes management.^[39]^ This shift not only improves clinical outcomes for patients with GDM but also streamlines workflows for healthcare providers, reducing time and costs associated with routine monitoring and data collection. As these technologies evolve, they hold the potential to further revolutionize GDM care, promising more proactive and patient-centered approaches.

## 7. Policy and public health strategies for managing GDM

### 7.1. Policy impact on diagnosis and treatment

Evaluating the role of governmental policies reveals that mandatory early screening for GDM legislated by some countries significantly boosts the early detection and timely intervention in GDM cases.^[40]^ Such proactive policy measures have been proven to reduce complications associated with pregnancy by facilitating early and effective treatment.

### 7.2. Public health promotion and education

Effective management of GDM is substantially supported by public health initiatives that focus on raising awareness and educating the population, particularly women of childbearing age.^[41,42]^ Organized by both governmental and non-governmental organizations, these initiatives deploy multiple channels such as media campaigns and community seminars to emphasize the importance of nutrition and physical activity. The objective is to not only decrease the prevalence of GDM but also empower individuals with the knowledge required for proactive management and self-care during pregnancy.

### 7.3. The role of interdisciplinary cooperation

The interdisciplinary approach in the management of GDM involves collaboration among obstetricians, endocrinologists, dietitians, and mental health professionals, forming a cohesive team that addresses the multifaceted needs of GDM patients.^[40,41,43]^ This model enhances the customization and accuracy of care, ensures efficient use of resources, and leads to better healthcare outcomes. Furthermore, it facilitates the integration of clinical practice with ongoing research, enhancing the development and application of innovative strategies for GDM prevention and treatment.

### 7.4. Comprehensive strategy and societal impact

Collectively, these approaches – rigorous policy implementation, educational outreach, and interdisciplinary collaboration – create a robust framework for reducing GDM incidence and improving maternal and fetal health outcomes.^[43]^ Such comprehensive strategies not only contribute to better health but also have a broader socio-economic impact by reducing healthcare costs associated with pregnancy complications and enhancing the overall quality of life for affected populations.

## 8. Future research directions and challenges

### 8.1. Research gaps and challenges

While substantial advancements have been made in understanding GDM, several critical areas require further exploration.^[12,44–47]^ A significant challenge lies in the early detection of GDM, especially in settings with limited resources. The reliance on glucose tolerance tests might overlook other potential biomarkers and predictive indicators essential for comprehensive screening. Furthermore, the heterogeneity in physiological responses among different ethnic and genetic populations highlights the need for more tailored treatment approaches. Additionally, there is a critical gap in longitudinal studies aimed at assessing the long-term effects of GDM on both mothers and their offspring.

### 8.2. Recommendations for future research

#### 8.2.1. Development of new diagnostic tools

Future studies should prioritize the development and validation of innovative biomarkers and noninvasive diagnostic tools. These advancements could facilitate earlier and more accurate detection of GDM, particularly beneficial in resource-poor environments.^[48–50]^

#### 8.2.2. Personalized treatment strategies

Research should expand to include more nuanced studies that consider ethnic, genetic, and socio-economic diversity among pregnant women. This approach would enable the formulation of customized management plans that are responsive to individual patient profiles.^[49–51]^

#### 8.2.3. Longitudinal and interdisciplinary studies

There is a pressing need for comprehensive long-term studies that monitor the health outcomes of GDM mothers and their children. These studies should investigate potential chronic conditions post-pregnancy, such as cardiovascular diseases and type 2 diabetes, to better understand the full spectrum of GDM’s impact.^[52]^

#### 8.2.4. Integration of modern technologies

The application of artificial intelligence and big data should be explored to revolutionize the management of GDM.^[52,53]^ Utilizing these technologies can lead to more precise predictions, enhanced monitoring, and optimized treatment protocols, ultimately improving patient outcomes.

## 9. Summary

GDM, as the predominant metabolic condition affecting pregnancies, demands rigorous management to mitigate maternal and neonatal complications. The advancements in prognosis for GDM-afflicted pregnancies are attributable to several factors: refined methodologies for identifying risk factors, the enhancement of screening protocols, and the implementation of targeted nutritional and exercise interventions coupled with timely pharmacotherapy. Despite these improvements, the management of GDM encounters ongoing challenges such as the need for uniform diagnostic standards, enhanced patient adherence to treatment regimens, and robust assessments of long-term health implications.

Future research directions in GDM are set to focus more intensely on personalized medical treatments. This will likely include leveraging genomic insights to anticipate risks associated with GDM and to tailor treatment efficacy, as well as employing advanced digital tools and artificial intelligence to refine disease monitoring and management strategies. Furthermore, the role of interdisciplinary studies will be crucial in spearheading innovations in GDM management, integrating expertise from endocrinology, nutrition, psychology, and public health. Such collaborative efforts are anticipated to not only refine the management of gestational diabetes with greater precision but also to establish preventive measures against the subsequent onset of type 2 diabetes and other metabolic disorders in postpartum women. These developments promise to elevate the standard of maternal and infant health globally.

## Author contributions

**Conceptualization:** Ya-ting Fan, Xin-hui Wang, Jing Cao.

**Data curation:** Ya-ting Fan, Jing Cao.

**Investigation:** Jing Cao.

**Methodology:** Ya-ting Fan, Xin-hui Wang, Qing Wang, Jing Cao.

**Project administration:** Jing Cao.

**Resources:** Ya-ting Fan, Xin-hui Wang, Qing Wang, Xiao-tong Luo.

**Supervision:** Jing Cao.

**Validation:** Ya-ting Fan, Xin-hui Wang, Qing Wang, Xiao-tong Luo, Jing Cao.

**Visualization:** Ya-ting Fan, Xin-hui Wang, Qing Wang, Xiao-tong Luo, Jing Cao.

**Writing – original draft:** Ya-ting Fan, Xin-hui Wang, Qing Wang, Xiao-tong Luo, Jing Cao.

**Writing – review & editing:** Ya-ting Fan, Xin-hui Wang, Qing Wang, Xiao-tong Luo, Jing Cao.
